# Proteomic Analysis Reveals That Placenta-Specific Protein 9 Inhibits Proliferation and Stimulates Motility of Human Bronchial Epithelial Cells

**DOI:** 10.3389/fonc.2021.628480

**Published:** 2021-05-28

**Authors:** Hai-Xia Wang, Xu-Hui Qin, Jinhua Shen, Qing-Hua Liu, Yun-Bo Shi, Lu Xue

**Affiliations:** ^1^ Institute for Medical Biology and Hubei Provincial Key Laboratory for Protection and Application of Special Plants in Wuling Area of China, College of Life Sciences, South-Central University for Nationalities, Wuhan, China; ^2^ Section on Molecular Morphogenesis, Eunice Kennedy Shriver National Institute of Child Health and Human Development (NICHD), National Institutes of Health (NIH), Bethesda, MD, United States

**Keywords:** PLAC9, iTRAQ, cell proliferation, cell cycle, cell migration, 16HBE

## Abstract

Placenta-specific protein 9 (PLAC9) is a putative secretory protein that was initially identified in the placenta and is involved in cell proliferation and motility. Bioinformatics analyses revealed that PLAC9 is repressed in lung cancers (LCs), especially lung adenocarcinomas, compared to that in the paired adjacent normal tissues, indicating that PLAC9 might be involved in the pathogenesis of pulmonary diseases. To investigate the potential role of PLAC9 in the abnormal reprogramming of airway epithelial cells (AECs), a key cause of pulmonary diseases, we constructed a stable PLAC9-overexpressing human bronchial epithelial cell line (16HBE-GFP-*Plac9*). We utilized the proteomic approach isobaric tag for relative and absolute quantification (iTRAQ) to analyze the effect of PLAC9 on cellular protein composition. Gene ontology (GO) and pathway analyses revealed that GO terms and pathways associated with cell proliferation, cell cycle progression, and cell motility and migration were significantly enriched among the proteins regulated by PLAC9. Our *in vitro* results showed that PLAC9 overexpression reduced cell proliferation, altered cell cycle progression, and increased cell motility, including migration and invasion. Our findings suggest that PLAC9 inhibits cell proliferation through S phase arrest by altering the expression levels of cyclin/cyclin-dependent kinases (CDKs) and promotes cell motility, likely *via* the concerted actions of cyclins, E-cadherin, and vimentin. Since these mechanisms may underlie PLAC9-mediated abnormal human bronchial pathogenesis, our study provides a basis for the development of molecular targeted treatments for LCs.

## Introduction

Lung diseases are one of the most common medical problems worldwide affecting the entire respiratory system, including the larynx, trachea, bronchi, and lungs (1–3). Lung diseases include, but are not limited to, asthma, bronchiectasis, bronchitis, chronic obstructive pulmonary disease (COPD), and lung cancers (LCs) (4–6). Lung diseases are a major public health issue, as they affect millions of people, and pose a social and economic burden for both the affected patients and the general population (7–11). Finding new and effective treatments is necessary to increase the life expectancy of patients and to improve their quality of life (12–14).

Although lung diseases differ in the underlying pathological mechanism, abnormal reprogramming of the airway epithelium is a common feature in most of them (15–23). Airway epithelial cells (AECs) express pattern recognition receptors, and orchestrate innate immune responses to potentially dangerous inhaled materials (15), thus contributing to the pathogenesis of several airway diseases, especially asthma and COPD. For example, an increase of apoptotic AECs has been reported in COPD patients (16). In addition, rhinovirus induces goblet cell hyperplasia in AECs *via* Notch signaling in COPD (17). Fibroblast-epithelial interactions may play an important role in the epithelial-mesenchymal transition (EMT) process in COPD (18). Moreover, AEC-derived cytokines are crucial for the pathogenesis of asthma (19), and airway epithelial barrier dysfunction contributes to asthma progression (20). In severe asthma, abnormal proliferation of epithelial cells in the respiratory system leads to airway remodeling, including the thickening of the epithelium and lamina reticularis (21). EMT is a critical developmental program involved in tumor metastasis in many types of cancers, including LC (22, 23). These studies demonstrated that pathological processes involving proliferation, migration, and apoptosis of AECs are associated with airway remodeling, airway barrier dysfunction, and EMT in several respiratory diseases. However, the underlying molecular mechanisms remain unclear.

Placenta-specific protein 9 (PLAC9) is a putative secretory protein that was initially identified in human placenta (24, 25). In a previous study, we found that PLAC9 could inhibit cell proliferation by altering cell cycle-related proteins phospho-c-Myc, cyclin B1, p21^Waf/Cip1^, and phospho-histone H3 (25). To investigate whether PLAC9 plays a role in lung epithelial cell function and pathology, we analyzed PLAC9 expression levels in lung cancer using various publicly available databases and found reduced PLAC9 expression in lung cancer. To systematically characterize the proteins that may be regulated by PLAC9, we constructed a stable cell line overexpressing PLAC9 derived from the human bronchial epithelial cell line 16HBE and analyzed the proteome using isobaric tag for relative and absolute quantification (iTRAQ). Bioinformatics analyses suggest that overexpression of PLAC9 in 16HBE alters cell physiological processes, especially cell proliferation, cell cycle, and cell migration. Consistently, we observed reduced cell proliferation, S-phase arrest, and increased cell migration and invasion in PLAC9 overexpressing cells. Taken together, our results suggest that PLAC9 might play a pathological role in lung-associated diseases, indicating that it might be a novel potential diagnostic and therapeutic target.

## Materials and Methods

### Cell Culture and Stable PLAC9-Overexpressing Clones

The human bronchial epithelial cell line 16HBE was purchased from Zhongqiaoxinzhou Biotech (Catalog Number: ZQ0001. Shanghai, China). 16HBE cells were cultured in Dulbecco’s modified Eagle’s medium (DMEM, Hyclone, Waltham, MA, USA) supplemented with 10% fetal bovine serum (FBS, ScienCell, San Diego, CA, USA) at 37°C under 5% CO_2_. A stable PLAC9-overexpressing cell line (16HBE-GFP-*Plac9*) and the corresponding stable control (16HBE-GFP) were custom-made by Genechem Co., Ltd, Shanghai, China.

### Protein Preparation and Digestion

Cells were randomly pooled into three groups. The three replicates were processed for iTRAQ analysis to identify differentially expressed proteins, as previously described with minor revisions (26, 27). Briefly, each sample was sonicated for 15 min in 500 μL SDT lysis buffer (4% sodium dodecyl sulfate (SDS) and 100mM Tris-HCl, pH 7.6). Samples were then incubated in water for 15 min at 95°C and centrifuged at 14,000 × g for 15 min. The supernatant was collected, and protein concentration was determined using the bicinchoninic acid (BCA) assay. Protein samples were then stored at -80°C until further analyses. 30 μL protein mixture was mixed with 1 M dithiothreitol (DTT) at a final concentration of 100 mM, and then incubated in water bath at 95°C for 5 min. After cooling to room temperature, the sample was mixed with 200 μL UA buffer (8 M urea and 150 mM Tris-HCl, pH 8.5), loaded onto an ultrafiltration filter (30-kDa cutoff, Sartorius, Germany), centrifuged at 12,500 × g for 25 min, and washed twice with UA buffer. Subsequently, 100 μL of iodoacetamide solution (100 mM iodoacetamide in UA buffer) was added to the filter, vortexed for 1 min at 600 rpm, incubated for 30 min at room temperature in the dark, and centrifuged at 12,500 × g for 25 min. The resulting filtrate was discarded. The filters were washed twice with 100 μL of UA buffer (12,500 × g, 15 min). Next, 100 μL of dissolution buffer (Applied Biosystems, Foster City, CA, USA) was added and centrifuged at 12,500 × g for 15 min. This step was repeated twice. Then, 40 μL of trypsin (Promega, Madison, WI, USA) buffer (5 μg trypsin in 40 μL dissolution buffer) was added to the filter. The filter was gently vortexed for 1 min at 600 rpm, incubated at 37°C for 16–18 h, transferred to a new tube, and centrifuged at 12,500 × g for 15 min. The digested peptides were collected with 20 μL of dissolution buffer, and the peptide concentration was measured using a Nanodrop 2000 spectrophotometer (Thermo Scientific, Wilmington, DE, USA) at 280 nm.

### iTRAQ Labeling and Analysis

A total of 100 μg of peptide mixture was labeled with iTRAQ reagents according to the manufacturer’s instructions (Applied Biosystems, Foster City, CA, USA). Triplicate 16HBE-GFP-*Plac9* samples were labeled with the reagents 114, 115, and 116, and triplicate 16HBE-GFP samples were labeled with the reagents 117, 118, and 121. The labeled samples were mixed and analyzed using an Agilent 1260 Infinity II HPLC system (Agilent Technologies, Palo Alto, CA, USA). The column was equilibrated with buffer A (10 mM HCOONH_4_, 5% (v/v) acetonitrile, pH 10.0), and the samples were separated in buffer B (10 mM HCOONH_4_, 85% (v/v) acetonitrile, pH 10.0) gradient as follows: 0% (v/v) for 25 min, 0–7% (v/v) for 5 min, 7–40% (v/v) for 35 min, 40–100% (v/v) for 5 min, and 100% (v/v) for 15 min at a flow rate of 1 mL/min. Approximately 36 samples were collected, lyophilized, dissolved in 0.1% formic acid (FA), and pooled into 10 fractions for further mass spectrometry analysis.

Each fraction was separated on an Easy nLC system (Thermo Fisher Scientific, San Jose, CA, USA). Buffer C consisted of 0.1% (v/v) formic acid in MilliQ water, and buffer D consisted of buffer C with 80% (v/v) acetonitrile. The samples were loaded *via* an autosampler onto an analytical column (Acclaim™ PepMap™ RSLC 50 μm × 15 cm, nano viper (Thermo Fisher Scientific, San Jose, CA, USA) and separated at a flow rate of 300 nL/min.

Peptide analysis was performed on a Q-Exactive Plus mass spectrometer (Thermo Fisher Scientific, San Jose, CA, USA) in positive ion mode for 120 min, with a selected mass range of 350–1,800 mass/charge (m/z). For the survey scan, the first-order mass spectrum resolving power was set to 70,000, the automatic gain control (AGC) target value was set to 3E6, and the first-order maximum ion injection (IT) time was 50 ms. The m/z ratios of polypeptide and polypeptide fragments were obtained according to the methods described in the following sections. Ten-fragment mass spectral (MS2) scans were collected after each full scan. The MS2 activation type was a higher-energy collisional dissociation (HCD). The isolation window was set at 2 m/z. The second-order MS resolution was 17,500 with a 1 µscan. The second-order maximum IT time was 45 ms. The normalized collision energy was 30 eV.

### Bioinformatics and Multivariate Analyses

Raw mass spectrometry (MS) data were derived from the RAW files. Database searches and quantitative analyses were performed using Mascot 2.6 and Proteome Discoverer 2.1 (Thermo Fisher Scientific, San Jose, CA, USA). Proteins were deemed to be differentially abundant if measured ratios exceeded a 1.2-fold change between the two sample groups analyzed and if the associated *p* value was below 0.05. The database used (Uniprot_HomoSapiens_161584_20180123) was downloaded from the UniProt website (http://www.uniprot.org) on 2018-01-23. The local sequence alignment software NCBI Basic Local Alignment Search Tool (BLAST 2.2.28+-win32.exe; http://blast.ncbi.nlm.nih.gov/Blast.cgi) was used to perform sequence alignment between the identified proteins and protein sequences in the UniProt database. The mapping function of the Blast2GO command line (www.geneontology.org; version go_201504.obo) was used to extract GO entries correlated with the sequences aligned for all the differentially expressed proteins identified. The webserver Kyoto Encyclopedia of Genes and Genomes (KEGG) Automatic Annotation Server (KAAS; http://www.genome.jp/tools/kaas/) was used to align the target proteins to the KEGG GENES database (28). The heatmap was generated using the online ClustVis tool (https://biit.cs.ut.ee/clustvis/) (29). The network of protein-protein interactions was mapped using the tool STRING 10.5, available online (http://string-db.org).

### Reverse Transcription and Quantitative Real-Time Polymerase Chain Reaction (RT-qPCR)

mRNA levels were determined using quantitative real-time PCR analysis using a QuantStudio^®^ 5 Real-Time PCR System (Applied Biosystems, Foster City, CA, USA) according to the manufacturer’s instructions. Briefly, total RNA was extracted from the cells using an RNA extraction kit (Bioteke, Beijing, China), and cDNA was synthesized using a cDNA synthesis kit (Thermo Fisher Scientific, San Jose, CA, USA). Then, 5 ng cDNA was amplified using SYBR Green Real-time PCR Master Mix (Toyobo, Osaka, Japan). The primers used are listed in **Table 1**. The PCR cycling conditions were as follows: 95°C for 30 s, 45 cycles of 95°C for 5 s, and 60°C for 30 s. PCR amplification was followed by melting curve analysis using the default program of the QuantStudio^®^ 5 Real-Time PCR machine (Thermo Fisher Scientific, San Jose, CA, USA). The mRNA expression levels of the genes of interest were calculated using the 2^-ΔΔCt^ method and normalized using the expression levels of glyceraldehyde 3-phosphate dehydrogenase (*GAPDH*).

**Table 1:** Sequences of primers used for Real-Time PCR.

Primer name	Sequences	Product sizes (bp)
Plac9-F	ATGGAGGAGATGGTAGAGAAGAC	241 bp
Plac9-R	CACATGAAGCTAAGGAAGGAAGT	
GAPDH-F	TGACTTCAACAGCGACACCCA	121 bp
GAPDH-R	CACCCTGTTGCTGTAGCCAAA	

### MTT Assay and Colony Formation

Cells were treated with 3-(4,5-Dimethylthiazol-2-yl)-2,5-diphenyltetrazolium bromide (MTT, Sigma, St Louis, MO, USA) for the cell proliferation assay, as previously described (25, 30). Briefly, the cells were seeded at 1,000 cells/well in a 96-well tissue culture plate. Then, cells were incubated with 20 µL of MTT reagent for 4 h at 37°C. After adding 200 µL dimethyl sulfoxide (DMSO) to each well, the absorbance was quantified at 490 nm at the time of DMSO administration, and 24, 48, 96, and 120 h later. In the colony formation assay, approximately 800 cells were plated and cultured in 6-well plates, fixed with ethanol, and stained with 0.5% crystal violet for 2 weeks. The colonies were imaged and quantified.

### Flow Cytometry Analysis

Flow cytometry was performed as described previously (25, 30). Briefly, approximately 10^6^ 16HBE cells were pelleted and fixed with 70% ethanol for 1 h at 4°C. After resuspending with staining buffer (2 mg/mL of propidium iodide (PI) and 10 mg/mL RNase in 1 × PBS), cell cycle distribution was assessed using a BD FACS Calibur Flow Cytometer (BD Biosciences, San Jose, CA, USA) according to the manufacturer’s instructions. For the apoptosis assay, approximately 10^6^ 16HBE cells were harvested, resuspended, and labeled with fluorescein isothiocyanate (FITC)-Annexin V and PI in the dark at room temperature for 15 min. Apoptotic cells were analyzed using a BD FACS Calibur flow cytometer according to the manufacturer’s instructions.

### Wound-Healing and Invasion Assays

Wound healing and invasion assays were performed as previously reported (31). 16HBE-*GFP*-*Plac9* or 16HBE-*GFP* clones (4 × 10^5^) per well were seeded in a 12-well plate with complete medium (DMEM containing 10% FBS). When cells achieved 90% confluence, a wound was created by scraping the cell layer with a sterile pipette tip. The floating cells were removed by replacing the culture medium. Digital images of the wounds were captured immediately after replacing the medium, and after 12 and 24 h. The healing rate was calculated by comparing the wound width at different time points to the starting point.

For the cell invasion assay, 2 × 10^5^ 16HBE-*GFP-Plac9* or 16HBE-*GFP* cells per well in 100 µL medium (DMEM supplemented with 1% FBS) were seeded in the top chambers of 24-well transwell plates (8.0 µm pore size; Corning Costar, Corning, NY, USA), and DMEM supplemented with 10% FBS was added to the bottom chamber. Cells were maintained at 37°C for 48 h. The cells on the top side of the filter were wiped out with a cotton swab, while those on the bottom side (those that had migrated) were fixed with 4% polyoxymethylene and stained with Giemsa staining solution (Yeasen, Shanghai, China). Five randomly selected fields per well were photographed, and the number of cells in five random fields per well was counted.

### Western Blot Analysis

Cells were collected and lysed in protein lysis buffer (Beyotime, Shanghai, China) supplemented with Phenylmethanesulfonyl fluoride (PMSF, Beyotime, Shanghai, China) and protease inhibitor cocktail (Bimake, Houston, TX, USA). Protein samples (20 μg per lane) were separated using 10% SDS-polyacrylamide gel electrophoresis (PAGE) and transferred to nitrocellulose (NC) membranes. The membranes were blocked with 5% nonfat dry milk and incubated with the corresponding antibodies (Cell Signaling Technology, Beverly, MA, USA) according to the manufacturer’s instructions. The protein bands were visualized using enhanced chemiluminescence (ECL)™ reagents (Thermo Fisher Scientific, San Jose, CA, USA). β-Actin was used as an internal control.

### Statistical Analysis

Differences between groups were analyzed for statistical significance using Student’s t-test. GO or KEGG enrichment analyses were performed using Fisher’s exact test. All experiments were repeated at least three times. Results are expressed as mean ± standard deviation (SD). Statistical significance was set at *p* < 0.05.

## Results

### PLAC9 Expression Is Repressed in Lung Cancers

**Figure 1:**
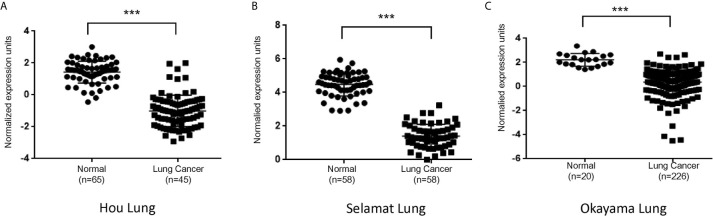
PLAC9 mRNA levels are reduced in lung cancer samples compared to normal tissue samples. PLAC9 mRNA expression in lung adenocarcinomas and paired adjacent normal tissues from three independent lung cancer studies (Hou Lung, Selamat Lung, and Okayama Lung, in **A–C**, respectively) were obtained from the Oncomine database and plotted individually. (***p value < 0.001).

To determine if PLAC9 expression is associated with LC, we screened the Oncomine database (www.oncomine.org). We observed that in three previously published LC cohorts (32–34), the expression of PLAC9 was significantly decreased in lung adenocarcinoma compared to paired adjacent normal tissues (**Figure 1**), implicating a role of PLAC9 in lung epithelial pathogenesis.

### Establishing a Cell Line Stably Expressing PLAC9 in Human Bronchial Epithelial Cell Line 16HBE

**Figure 2:**
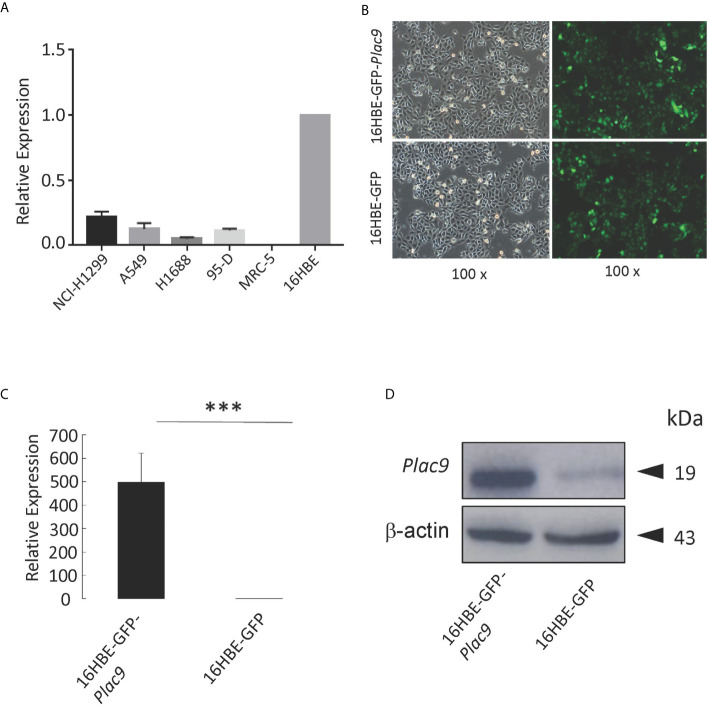
Construction of stable PLAC9-expressing 16HBE cells *via* lentiviral transduction. **(A)** Relative PLAC9 mRNA expression levels in the human lung cell lines NCI-H1299, A549, H1688, 95-D, MRC-5, and 16HBE. **(B)** 16HBE was used to generate stable cell lines expressing GFP (16HBE-*GFP*) or both GFP and PLAC9 (16HBE-*GFP*-*Plac9*). Detection of GFP in stable cell lines 16HBE-*GFP*-*Plac9* and 16HBE-GPF using fluorescence microscopy (green, GFP; 100× amplification). Left panel: bright field. Right panel: dark field. **(C)** Reverse transcription quantitative PCR analysis of PLAC9 expression levels shows overexpression of PLAC9 mRNA in the stable cell line 16HBE-*GFP*-*Plac9* compared to the control line 16HBE-*GFP* (***p value < 0.001). **(D)** Western blot analysis shows overexpression of PLAC9 protein in the stable cell line 16HBE-GFP-*Plac9* compared to the control.

To investigate the role of PLAC9 in LC, we evaluated the mRNA expression levels of *PLAC9* in several of lung carcinoma cell lines, including NCI-H1299, A549, H1688, 95-D, MRC-5 as well as in the human bronchial epithelial cell line 16HBE (**Figure 2A**). The levels of *PLAC9* mRNA were almost undetectable in human lung fibroblast cells MRC-5, and the rest of the cell lines had very low levels of *PLAC9* mRNA. The expression level of *PLAC9* mRNA in 16HBE cells was significantly higher than that in the lung cancer cell lines NCI-H1299, A549, H1688, and 95-D. The mRNA expression pattern of *PLAC9* in normal lung cells compared with lung carcinoma cells was consistent with the expression of *PLAC9* in normal lung tissue compared with lung carcinoma, as shown in **Figure 1**.

To further explore the role of PLAC9 in human bronchial epithelial pathogenesis, we constructed a stable PLAC9-overexpressing cell line (16HBE-*GFP*-*Plac9*). For this purpose, we transfected the lentiviral *Plac9* vector, which expresses both *GFP* and *Plac9* under the control of the constitutive CMV promoter, into 16HBE cells. In addition, we generated a control line stably expressing only GFP, 16HBE-GFP, as previously described (25). **Figure 2B** shows a representative photograph of the stable cell lines generated (16HBE-*GFP*-*Plac9* and 16HBE-GFP). Further qRT-PCR and western blot analyses showed that the 16HBE-*GFP-Plac9* cells had significantly increased mRNA and protein levels of PLAC9 compared to the control 16HBE-*GFP* cells (**Figures 2C, D**).

### iTRAQ Analysis of PLAC9-Regulated Proteins

**Figure 3:**
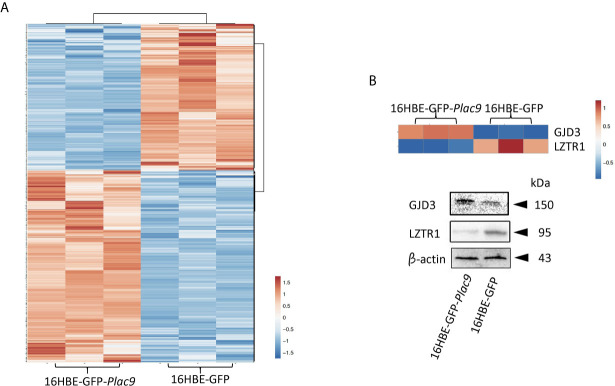
Global identification of proteins regulated by PLAC9 overexpression. **(A)** Heatmap of the 714 differentially expressed proteins identified by iTRAQ in the PLAC9-overexpressing line compared to the control line stably transfected with GFP-expression vector. Three replicates are presented for each cell line. Bar color represents a logarithmic scale from -1.5 to 1.5. **(B)** Independent validation of two differentially expressed proteins (GJD3 and LZTR1) identified *via* iTRAQ using western blot analysis. Total proteins were isolated from the two cell lines (16HBE-GFP-*Plac9* and 16HBE-GFP) for western blot analyses with antibodies against GJD3, LZTR1, and the control was β-actin.

To investigate the changes in protein expression caused by PLAC*9* overexpression, iTRAQ quantitative proteomic technology was applied to analyze the protein samples from 16HBE-*GFP*-*Plac9* and control 16HBE-*GFP* cells. The MS proteomics data were deposited to the ProteomeXchange Consortium (http://proteomecentral.proteomexchange.org) *via* the iProX partner repository (35) with the dataset identifier PXD019147. A total of 279,431 spectra were generated, of which 125,694 spectra matched known peptides. Ultimately, 69,296 peptide fragments were identified, among which 6,841 unique proteins were detected and quantified. The peptide/protein distribution-based ion score, molecular weight, isoelectric point, peptide length, protein sequence coverage, peptide count, and ratio of expression levels between the two cell lines were analyzed and found to be consistent with a technically successful and reliable iTRAQ experiment (**Figure S1**). Considering a threshold value of fold change ≥ ± 1.2 and *p* < 0.05, 714 proteins were found to have significantly different levels that those in *PLAC9*-overexpressing 16HBE cells (**Table S1**, see also the volcano plot in **Figure S2**). Among them, 405 proteins were upregulated, whereas 309 proteins were downregulated (**Table S1**). Hierarchical cluster analysis of the proteins that differed in abundance in the three replicates of each cell line showed highly reproducible patterns (**Figure 3A**). The validity of iTRAQ was independently confirmed using western blot analysis of two of the proteins that presented the most significant differences. The proteins analyzed were gap junction protein delta 3 (GJD3), which was the most upregulated protein (18.4-fold increase, *p* value < 0.001) and leucine zipper like transcription regulator 1 (LZTR1), which was the most downregulated protein (0.21-fold decrease, *p* value < 0.001) (**Figure 3B**).

### Bioinformatics Analyses of the PLAC9 Regulated Proteins

**Figure 4:**
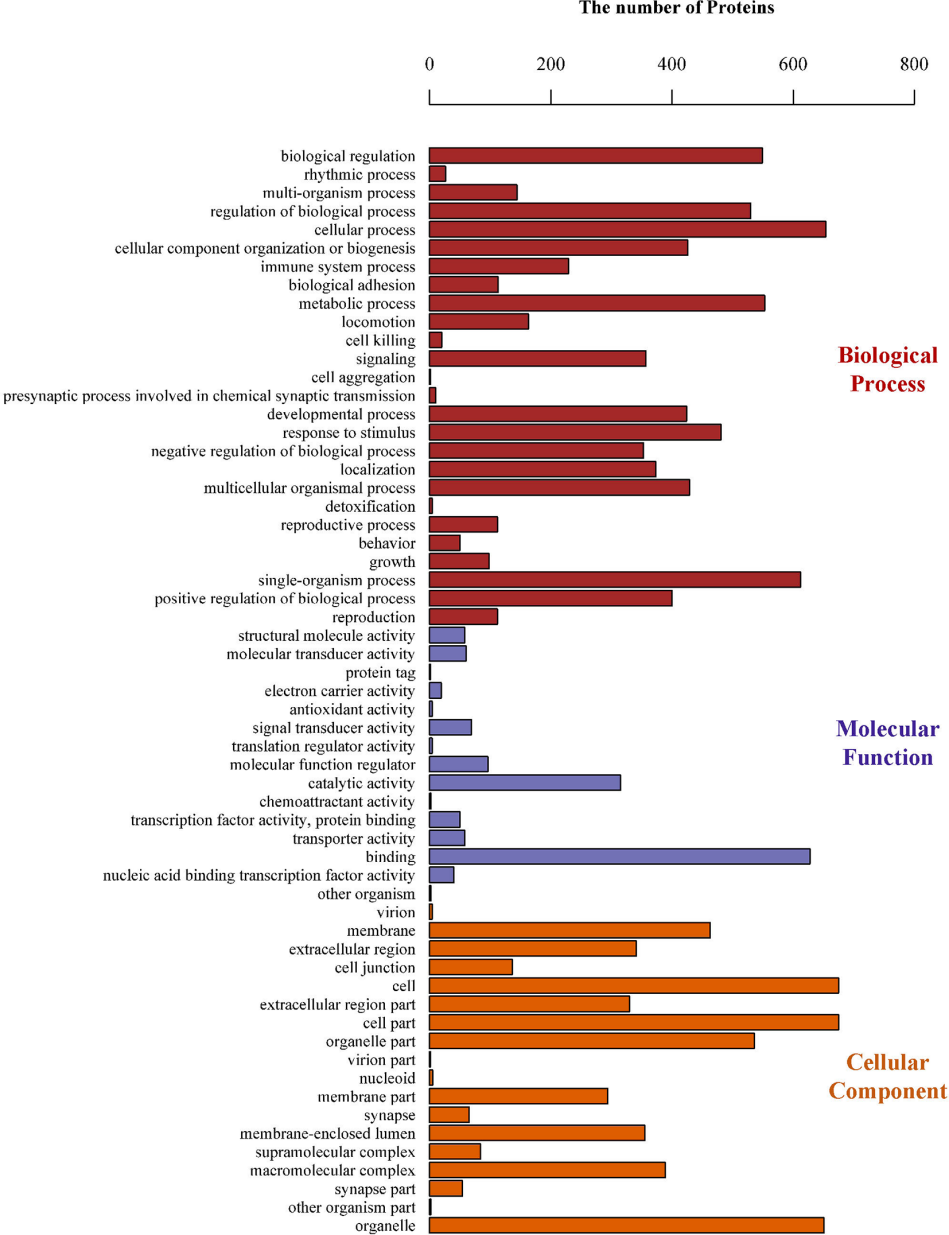
Gene Ontology (GO) classifications of the differentially expressed proteins. The number of proteins involved in each of the biological process, cellular component, and molecular function terms, which could be classified into 26, 15, and 18 subcategory groups, respectively.

To gain insight into the functions of the proteins that were altered by PLAC9 overexpression, the differentially expressed proteins were categorized into three groups (biological process, molecular function, and cellular component) based on GO analysis. The differentially expressed proteins covered a wide range of biological processes, molecular functions, and cellular components, which could be classified into 26, 15, and 18 subcategory groups, respectively (**Figure 4**). The largest group within the biological process category was that of cellular processes (654/668), followed by single-organism processes (612/668) and metabolic processes (553/668). Binding (628/662) and catalytic (315/662) activity were the most common categories for molecular function. The cellular component functions of these proteins were mainly related to the cell (675/685), organelles (651/685), membranes (463/685), and macromolecular complexes (389/685). The top 30 enriched GO categories are listed in **Table 2**, and the total enriched GO categories are listed in **Table S2**. These results showed that the predominant functions of the differentially expressed proteins were cellular processes, particularly cell migration.

**Table 2:** Top 30 enriched GO terms among the differentially expressed proteins.

GO-ID	Term	Category	#DIFF	%DIFF	P-Value
**GO:0032502**	developmental process	P	424	59.38	1.67E-10
**GO:0044767**	single-organism developmental process	P	410	57.42	2.7E-09
**GO:0048869**	cellular developmental process	P	316	44.26	6.82E-09
**GO:0048856**	anatomical structure development	P	394	55.18	8.46E-09
**GO:0048731**	system development	P	345	48.32	8.66E-09
**GO:0042221**	response to chemical	P	338	47.34	9.42E-09
**GO:0048513**	animal organ development	P	280	39.22	1.13E-08
**GO:0009888**	tissue development	P	190	26.61	0.000000012
**GO:0006952**	defense response	P	164	22.97	1.25E-08
**GO:0048646**	anatomical structure formation involved in morphogenesis	P	110	15.41	1.31E-08
**GO:0022613**	ribonucleoprotein complex biogenesis	P	28	3.92	3.94E-08
**GO:0030154**	cell differentiation	P	298	41.74	3.96E-08
**GO:0009605**	response to external stimulus	P	199	27.87	5.43E-08
**GO:0004872**	receptor activity	F	60	8.40	8.28E-08
**GO:0060089**	molecular transducer activity	F	60	8.40	8.28E-08
**GO:0007275**	multicellular organism development	P	367	51.40	8.76E-08
**GO:0032501**	multicellular organismal process	P	429	60.08	0.000000091
**GO:0006954**	inflammatory response	P	59	8.26	0.000000446
**GO:0042254**	ribosome biogenesis	P	18	2.52	0.000000528
**GO:0044707**	single-multicellular organism process	P	399	55.88	0.000000644
**GO:0000786**	nucleosome	C	13	1.82	0.000000655
**GO:0050793**	regulation of developmental process	P	212	29.69	0.000000839
**GO:0051240**	positive regulation of multicellular organismal process	P	149	20.87	0.000000853
**GO:0065008**	regulation of biological quality	P	317	44.40	0.000000927
**GO:0050691**	regulation of defense response to virus by host	P	39	5.46	0.0000012
**GO:0035425**	autocrine signaling	P	9	1.26	0.00000152
**GO:0006955**	immune response	P	152	21.29	0.00000159
**GO:0071944**	cell periphery	C	288	40.34	0.00000226
**GO:0016477**	cell migration	P	145	20.31	0.00000233
**GO:0051239**	regulation of multicellular organismal process	P	234	32.77	0.00000249

P, represents biological process. C, represents cellular component. F represents molecular function. #DIFF represents the number of differentially expressed proteins involved in certain GO term. %DIFF represents the ratio of #DIFF to the total differentially expressed proteins. The data was sorting by FDR.

To identify the biological pathways up- or down-regulated in PLAC9-overexpressing 16HBE cells, we conducted KEGG pathway-based analysis for the differentially expressed proteins and obtained 316 maps. Predictions for the most differentially expressed proteins suggest that they are involved in 20 pathways (**Table 3**), all of which are listed in **Table S3**. Among these pathways, RNA transport, RNA polymerase, mRNA surveillance pathways, all of which regulate gene expression, and the cell adhesion molecule (CAM) pathway, which regulates cell proliferation and migration, are notable. For example, among the regulated pathways, we found that CDK2 (cyclin-dependent kinase 2) was downregulated (0.9-fold change, *p* value < 0.05), while E-cadherin was upregulated (1.1-fold change, *p* value < 0.05), which indicated that overexpression of PLAC9 might influence cell proliferation and migration.

**Table 3:** Top 20 enriched KEGG pathways among the differentially expressed proteins.

MapID	MapName	Differentially expressed proteins
**ko05322**	Systemic lupus erythematosus	↑ HIST3H3, HIST1H3A, HIST2H2AB, HIST2H3PS2, H3F3A, HIST1H3D, H2AFX, FLJ94402, HIST1H4A
		↓ ACTN1, HEL-S-62p, ACTN4, C9
**ko05146**	Amoebiasis	↑ SERPINB3, SERPINB4, SERPINB13, SERPINB9, SERPINB6, PRKACA, PIK3CB
		↓ FN1, ACTN1, LAMB3, LAMC2, LAMA3, ACTN4, C9
**ko03013**	RNA transport	↓ EEF1A2, NUP210L, TACC3
**ko03010**	Ribosome	↑ MRPL23, MRPL10
		↓ MRPS14
**ko05034**	Alcoholism	↑ HIST3H3, HIST1H3A, HIST2H2AB, HIST2H3PS2, H3F3A, HIST1H3D,H2AFX, Histone H2B, GNAI3, HIST1H4A,
		↓ P36873, HDAC
**ko04610**	Complement and coagulation cascades	↑ CLU, SERPINB2, SERPINE1
		↓ HEL-S-62p, RAB1A, C9, F3
**ko04260**	Cardiac muscle contraction	↑ COX6A1, COX2, COX5B, COX7C, COX6B1, COX7A2
		↓ TPM4, HEL-S-265, TPM1
**ko00240**	Pyrimidine metabolism	↑ POLR3K, POLR2I, TP, POLA2, FLJ92093, RNAP, POLR2L, NT5C3B
		↓ CANT1, UPP1, POLR2G, FLJ54187, UPRT, HCG23833, UCK2, NT5E
**ko04750**	Inflammatory mediator regulation of TRP channels	↑ MAP2K6, CD74-Ntrk1, TRPV4, PRKACA, PIK3CB
		↓ PLA2s, PRKCH, PPP1CC, PKCϵ
ko00120	Primary bile acid biosynthesis	↑ CYP46A1, FLJ93299
		↓ CYP7B1
ko00740	Riboflavin metabolism	↑ ACP1
		↓ RFK, ACP2
ko03020	RNA polymerase	↑ POLR3K, POLR2I, FLJ92093, RNAP, POLR2L
		↓POLR2G, FLJ54187
ko04913	Ovarian steroidogenesis	↑ PRKACA
		↓ LDLR, PLA2s, PTGS2
ko03015	mRNA surveillance pathway	↑ mRNA-capping enzyme
		↓ PPP1CC
**ko03008**	Ribosome biogenesis in eukaryotes	↓ RIOK2, DKFZp686O2396
**ko04144**	Endocytosis	↑ RUFY1, FOLR1, WASHC1
		↓ LDLR, SPG20, WASHC2C, FLJ33900, SNX32, CHMP4C, FLJ96001
**ko05321**	Inflammatory bowel disease (IBD)	↑ STAT1, STAT
		↓ TLR5, IL1A
**ko00760**	Nicotinate and nicotinamide metabolism	↑ NNMT, NADK2, NT5C3B
		↓ NT5E
**ko04630**	Jak-STAT signaling pathway	↑ FHL1, STAT1, CREBBP, OSMR, FLJ12419, STAT, PIK3CB
		↓ BCL2L1
**ko04514**	Cell adhesion molecules (CAMs)	↑ ITGA4, FLJ77845, ALCAM, MPZL1, NECTIN1, GLG1, FLJ54854
		↓ CD274

Up and down arrows indicated increased and decrease levels in Plac9-overexpressed 16HBE cell line compared to the GFP-16HBE cell line, respectively.

**Figure 5:**
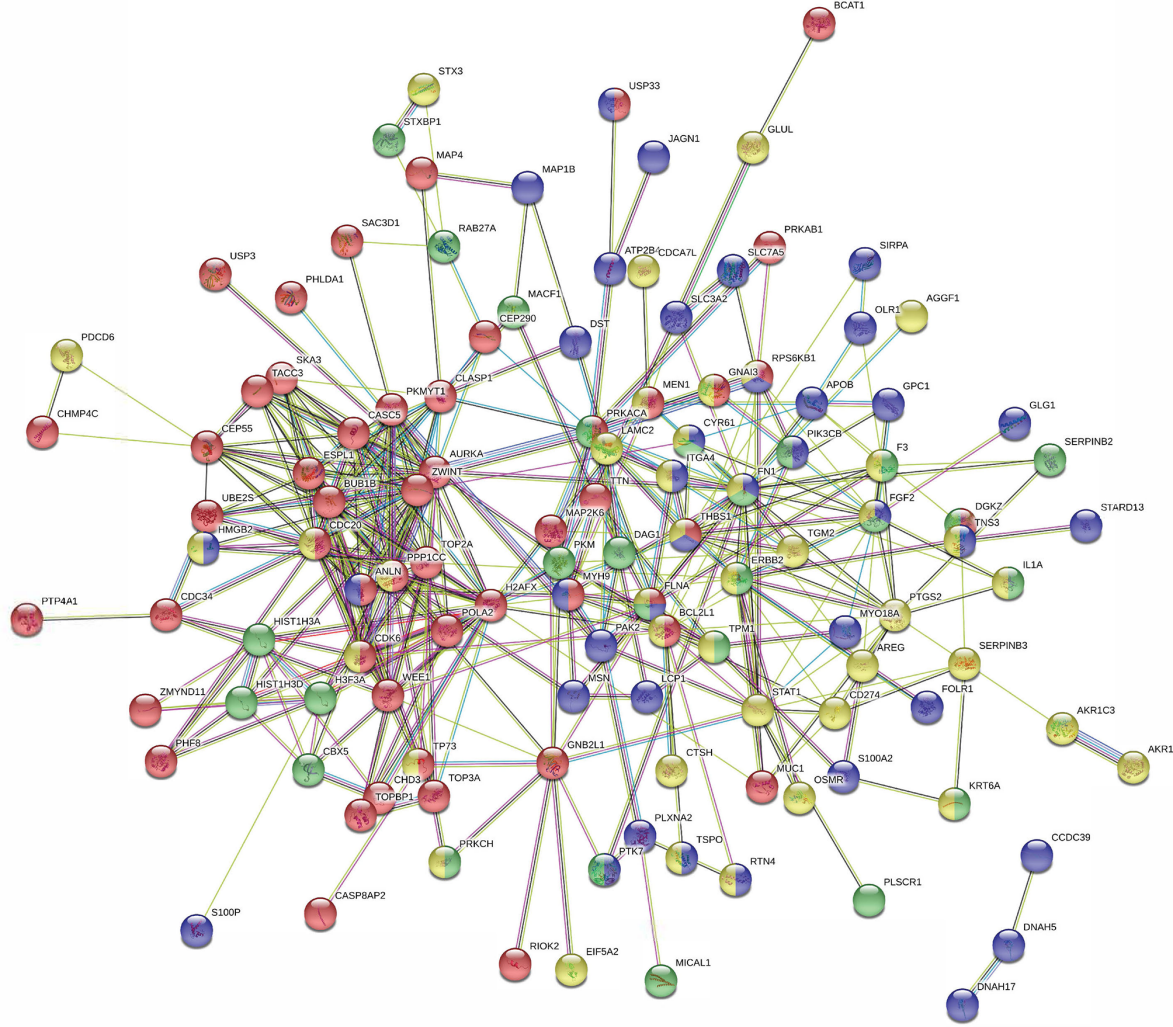
STRING protein-protein interaction network for the 138 proteins involved in cell proliferation, cell cycle, and cell migration. Network of interactions among 138 regulated proteins involved in cellular processes. Network nodes represent the proteins. A protein/node may be filled with up to four different colors. Yellow represents proteins involved in cell proliferation, red represents proteins involved in cell cycle, green represents proteins involved in wound healing, and blue represents proteins involved in cell motility. The minimum required interaction score was medium confidence (0.400). The structure previews of proteins are displayed inside the nodes. All the disconnected nodes in the network were hidden. The details of this figure are presented in **Table S5**.

To further explore these hypotheses, the protein-protein interaction network was analyzed using the publicly available program STRING. The results are shown in **Figure S3** and **Table S4**. A total of 714 differentially expressed proteins determined from iTRAQ were analyzed using the molecular interaction tool. A total of 587 interactive proteins were identified. One quarter of the proteins identified were related to cell proliferation, cell cycle, and cell motility (**Figure 5** and **Table S5**). The protein interaction network indicated that PLAC9 might play an important role in several cellular processes, especially cell proliferation, cell cycle, and cell migration, further supporting the potential role of PLAC9 in cell proliferation and motility.

### Effect of PLAC9 on Cellular Processes Including Cell Proliferation, Cell Cycle, and Cell Migration

**Figure 6:**
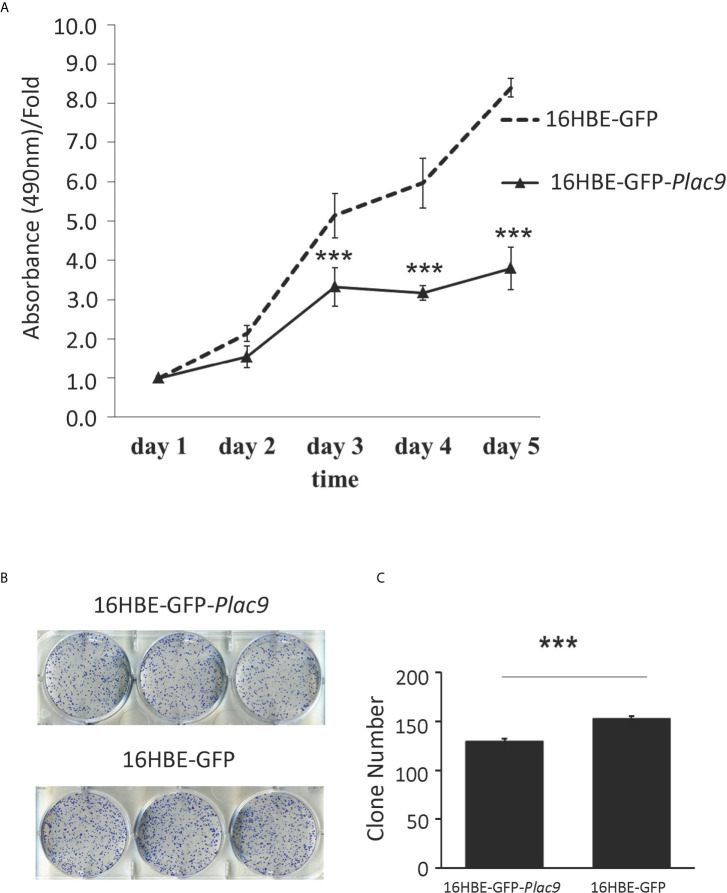
Overexpression of PLAC9 inhibits cellular proliferation and colony formation. **(A)** Overexpression of PLAC9 inhibits cell proliferation. 16HBE-GFP-*Plac9* and the control line 16HBE-GFP were cultured, and cell number was determined daily for 5 days, by measuring the absorbance of the cell culture at 490 nm with the value at staring point (day 1) set to 1. **(B, C)** Overexpression of PLAC9 inhibits colony formation. 16HBE-GFP-*Plac9* and the control line 16HBE-GFP were cultured for 2 weeks to assay for colony formation **(B)** and the number of colonies/plate were quantified **(C)**. (***p < 0.001).

To investigate the role of PLAC9 in cell proliferation, MTT and colony formation assays were performed using 16HBE-*GFP*-*Plac9* and 16HBE-*GFP* cell lines. The results of MTT assay showed that the proliferative capacity of 16HBE-*GFP*-*Plac9* cells was significantly lower than that of 16HBE-*GFP* cells (**Figure 6A**). Furthermore, *in vitro* colony formation assays demonstrated that the frequency of colony formation of 16HBE-*GFP*-*Plac9* cells was significantly lower than that of 16HBE-*GFP* cells (**Figures 6B, C**). These results indicated that PLAC9 plays an inhibitory role in 16HBE proliferation.

**Figure 7:**
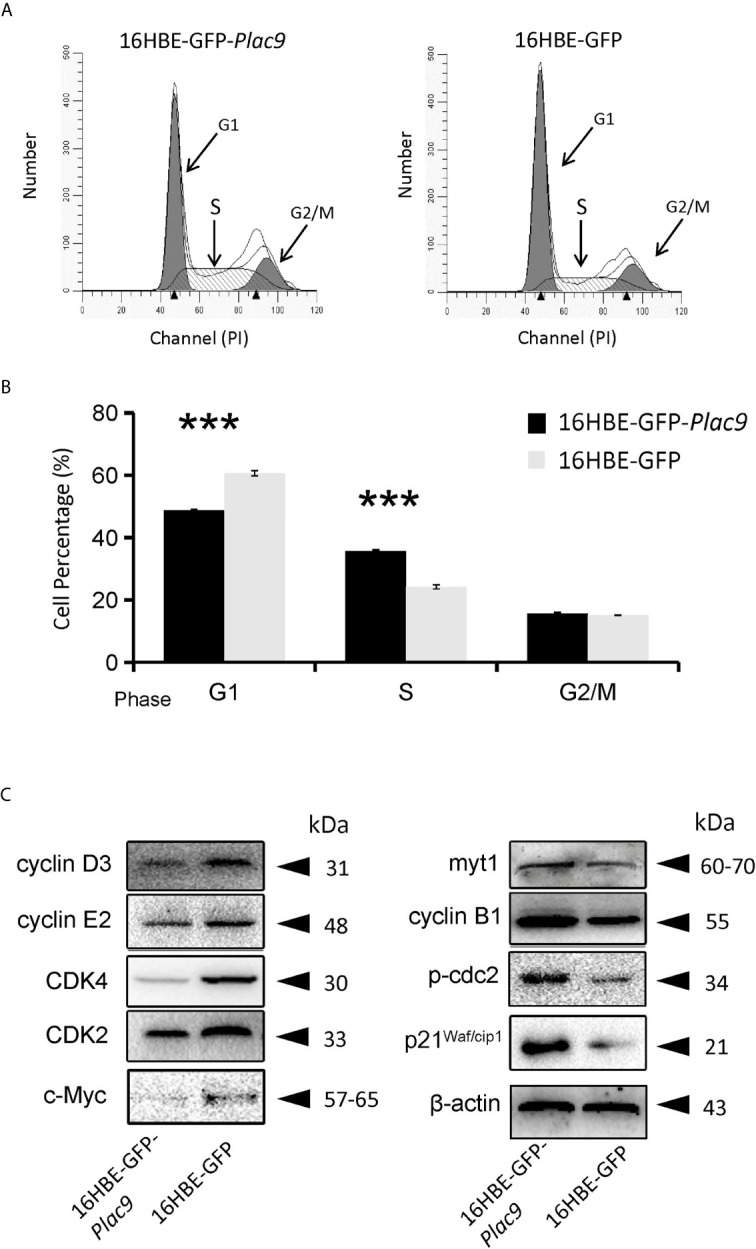
Overexpression of PLAC9 alters cell cycle distribution. **(A, B)** Overexpression of PLAC9 increases the cell population in S phase, while reducing the number of cells in G1 phase. 16HBE-GFP-*Plac9* and control 16HBE-GFP cells were incubated with PI and analyzed using flow cytometry **(A)** and quantified **(B)**. (***p < 000.1). **(C)** Plac9 overexpression alters the expression of cell cycle proteins The total proteins of 16HBE-GFP-*Plac9* and the control line 16HBE-GFP were isolated for western blot analysis of cell cycle-related proteins. Representative blots from three experiments with similar results are shown.

According to the iTRAQ data, the key regulator of the G1-S transition CDK2 was downregulated (0.9-fold, *p* value < 0.05). Consistently, flow cytometry showed that overexpression of PLAC9 altered the cell cycle distribution of 16HBE cells (**Figures 7A, B**). The number of cells in G1 phase was reduced, while the number of cells in S phase was significantly increased when PLAC9 was overexpressed. Furthermore, western blot analysis showed that the level of proteins promoting cell cycle progression, such as c-Myc, cyclin D3, cyclin E2, CDK2, and CDK4 were decreased in cells overexpressing PLAC9. Meanwhile, the protein levels of cell cycle inhibitors such as p21^Waf/cip1^ and myt1 were increased in PLAC9-overexpressing cells (**Figure 7C**). In addition, M-phase entry repressors, including cyclin B1 and p-cdc2, were also increased. In contrast, overexpression of PLAC9 had no effect on apoptosis (data not shown).

**Figure 8:**
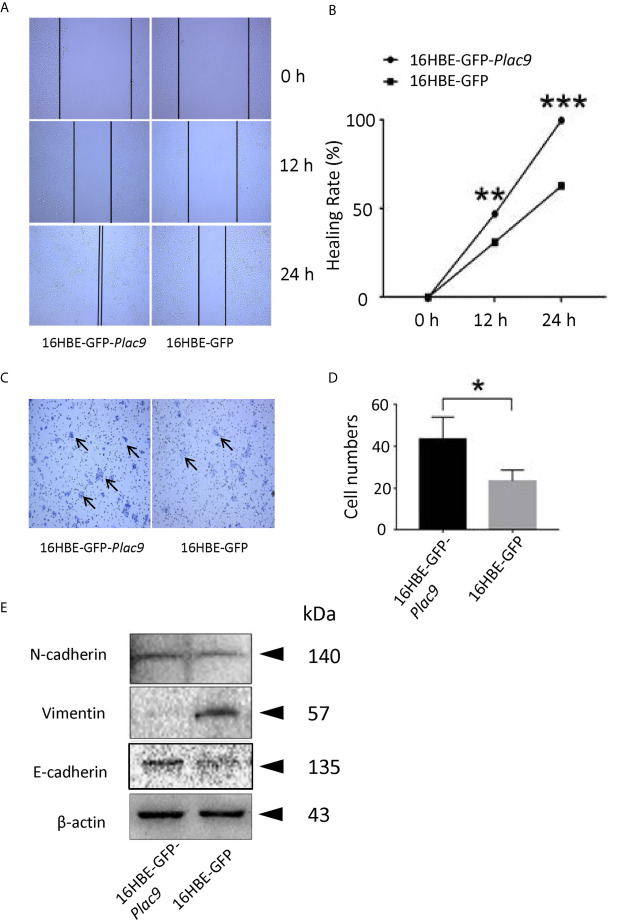
Overexpression of PLAC9 enhances cell migration. **(A, B)** PLAC9 increases cell motility in a wound-healing assay. PLAC9 overexpressing stable cell line (16HBE-GFP-*Plac9)* and the control line 16HBE-GFP were cultured for 0, 12, or 24 h in the wound-healing assay **(A)**. The wound healing rate for 16HBE-GFP-*Plac9* was 42.01 ± 5.05% and 85.93 ± 7.56% at 12 h and 24 h, respectively, while for 16HBE-GFP, it was 29.28 ± 1.76% and 56.64 ± 5.75% at 12 h and 24 h, respectively **(B)**. (*p < 0.05, **p < 0.01, ***p < 0.001). **(C, D)** PLAC9 increases cell invasion. PLAC9 overexpression stable cell line (16HBE-GFP-*Plac9)* and the stable control line (16HBE-GFP) were cultured for 48 h in the transwell cell invasion assay **(C)** and quantified **(D)**. (*p < 0.05, **p < 0.01, ***p < 0.001). **(E)** PLAC9 alters the expression of cell migration-related proteins Total proteins of PLAC9 overexpressing stable cell line (16HBE-GFP-*Plac9)* and the control line (16HBE-GFP) were isolated for western blot analysis.

As indicated above, the iTRAQ data suggest that PLAC9 affects cell migration. To explore this hypothesis, we performed two cell motility assays (wound healing and cell invasion assay) as previously described elsewhere (36). As shown in **Figures 8A, B**, the wound-healing assay showed that wound closure after 48h in 16HBE-*GFP*-*Plac9* cells was more pronounced than that in 16HBE-*GFP* cells, indicating that PLAC9 overexpression strongly enhanced cell migration. Next, we used a Transwell^®^ chamber to perform the cell invasion assay (**Figures 8C, D**). The number of invading cells was significantly higher in 16HBE-GFP-*Plac9* cells than in control cells. The results indicate that overexpression of PLAC9 markedly increased cellular motility.

It is well known that E-cadherin, N-cadherin, and vimentin are key regulators of embryonic development, organ morphogenesis, and tumor growth (37, 38), and are involved in epithelial cell motility *via* EMT (39–41). In PLAC9-overexpressing 16HBE cells, E-cadherin levels were significantly higher than those in the control, which was consistent with the iTRAQ data (**Figure 8E**), while vimentin was inhibited in PLAC9-overexpressing 16HBE cells. N-cadherin levels were not significantly different between the two cell lines analyzed. Taken together, these results indicated that the overexpression of PLAC9 facilitated cell motility despite increased E-cadherin and reduced vimentin protein levels.

## Discussion

Abnormal physiological processes of the airway epithelium are one of the key features of several lung diseases. Here, we provide evidence for the role of a putative secretory protein, PLAC9, in the pathophysiology of human airway epithelial cells. First, we observed decreased expression of PLAC9 in various LCs, especially lung adenocarcinoma, in samples compiled in a publicly available database. Notably, our global proteomic analysis of PLAC9*-*regulated proteins and cell culture studies support the role of PLAC9 in inhibiting cell proliferation and promoting cell migration.

Furthermore, the proteomic technique iTRAQ was employed to identify PLAC9 regulated proteins. A total of 714 proteins were identified, of which approximately one quarter are related to cell proliferation, cell cycle, and cell motility. Consistently, MTT and colony formation experiments provided clear evidence that the overexpression of PLAC9 inhibits cell proliferation. This result was consistent with a previous study, in which we showed that PLAC9 overexpression inhibits proliferation of L02 cells, a human embryo liver cell line (25). Furthermore, cell cycle distribution data showed that overexpression of PLAC9 in 16HBE cells increased the percentage of cells in S phase and decreased the percentage of cells in G1 phase. Mechanistically, PLAC9 appears to induce cell cycle arrest at the S phase by regulating the levels of cell cycle-associated proteins, including cyclins and CDKs.

Cyclins and CDKs are pivotal for cell cycle progression (33). Among them, the complexes cyclin D/CDK4 and cyclin E/CDK2 are critical for G1-S progression (42). Consistently, downregulation of cyclin D3, cyclin E2, CDK2, and CDK4 in the cell line 16HBE-*GFP*-*Plac9* was associated with the accumulation of cells in S phase. This result is in line with earlier studies showing that S phase arrest is associated with downregulation of the complexes cyclin D/CDK4 and cyclin E/CDK2 (43, 44).

Activation of the complex cyclin B1/cdc2 is a pivotal step in mitosis entry, mainly mediated by dephosphorylation of cdc2 (45). Myelin transcription factor 1 (Myt1), a member of the Wee kinase family, phosphorylates cdc2 to prevent mitosis initiation (46). In 16HBE-*GFP*-*Plac9* cells, accumulation of cells in S phase was accompanied by the upregulation of p-Cdc2, Myt1, and cyclin B1, suggesting that PLAC9 induced cdc2 phosphorylation, which prevents mitosis entry and facilitates S phase arrest.

p21^Waf/cip1^ protein is a cell cycle inhibitor that plays an important role in cell growth arrest (47, 48). The proto-oncogene c-Myc suppresses p21^Waf1/cip1^ expression, and downregulation of c-Myc induces S phase arrest and inhibits cell proliferation (49, 50). In 16HBE-GFP-*Plac9* cells, c-Myc and its transcriptional target, p21^Waf/cip1^ were down- and up-regulated, respectively, likely contributing to S phase arrest and reduced cell proliferation.

Taken together, these results indicate that PLAC9 induces S phase arrest *via* the inactivation of cyclin E2/CDK2 and cyclin D3/CDK4 complexes, myt1-induced cyclin B1/cdc2 phosphorylation, and c-Myc downregulation. The negative effect of PLAC9 on cell cycle progression suggests that PLAC9 may be a potential tumor suppressor.

Our cell motility assays showed that overexpression of PLAC9 facilitated cell migration. Western blotting analysis showed that E-cadherin was upregulated in 16HBE-GFP-*Plac9* cells, whereas vimentin was downregulated. Normally, increased cell migration relies on the loss of E-cadherin expression and the acquisition of vimentin expression (51, 52). However, recent evidence suggests that besides classical cadherins, cell cycle proteins have several non-canonical roles in cell migration, in addition to their well-established functions in driving cell proliferation (53). For instance, long non-coding RNA p53-inducible cancer-associated RNA transcript 1 (PICART1) stimulates cell migration in tumor cells by decreasing c-Myc and increasing p21^Waf/Cip1^ expression levels (54). High CDK2 activity can phosphorylate breast cancer metastasis suppressor 1 (BRMS1) and suppress cell migration (55). Cell migration of BMAL1 (brain and muscle aryl hydrocarbon receptor nuclear translocator-like 1)-knockdown glioblastoma cells was elevated, accompanied with upregulation of cyclin B1 (56). Taken together, PLAC9 overexpression-induced cell motility probably relies on the combination of altered expression of CDK2, cyclin B1, c-Myc, and p21^Waf/cip1^, despite the unexpected changes in E-cadherin and vimentin levels.

## Conclusions

This study provides a global protein regulation profile underlying the molecular function of PLAC9 in the human airway epithelium. Of the 714 differentially expressed proteins in the PLAC9-overexpressed 16HBE cell line, a quarter of them were associated with cell proliferation, cell cycle, and cell motility. To support this, we provided experimental evidence showing that PLAC9 inhibits cell proliferation, accompanied by S-phase arrest, and promotes cell migration. To our knowledge, this is the first study to investigate the mechanism by which PLAC9 affects the proliferation and migration of human bronchial epithelial cells. Our findings suggest that PLAC9 may be involved in lung diseases by regulating cell proliferation and migration.

However, only one type of lung-derived cell line was used in this study. More lung cancer cell lines and clinical samples should be examined to reach a comprehensive conclusion. Further studies are necessary to fully understand the regulatory mechanisms of PLAC9 in the abnormal reprogramming of the airway epithelium *in vitro* and *in vivo*. Meanwhile, the upstream molecular mechanism involved in the reduction of PLAC9 expression in LCs should be explored in further studies.

## Data Availability Statement

The datasets presented in this study can be found in online repositories. The names of the repository/repositories and accession number(s) can be found below: [ProteomeXchange, PXD019147].

## Author Contributions

JS, Q-HL, Y-BS, and LX conceived and designed the experiments. X-HQ and H-XW performed the experiments. X-HQ, H-XW, and LX analyzed the data and generated the figures. LX and Y-BS wrote the manuscript. All authors contributed to the article and approved the submitted version.

## Funding

This project was supported by the Fund for Key Laboratory Construction of Hubei Province (Grant No. 2018BFC360), the National Natural Science Foundation of China (Grant No. 31101047 to LX), the Natural Science Foundation of Hubei Province, China (Grant No. 2018CFB594 to LX), and the China Scholarship Council (grant number 201808420069 to LX). YBS was supported by the NICHD intramural program NICHD NIH, USA). The funding body had no role in the design of the study, collection, analysis, and interpretation of data, or in writing the manuscript.

## Conflict of Interest

The authors declare that the research was conducted in the absence of any commercial or financial relationships that could be construed as a potential conflict of interest.

## Supplementary Material

Supplementary Figure 1 Global characteristics of peptide properties identified in iTRAQ. **(A)** Normalized Mascot ion score distributions. **(B)** Normalized molecular weight distribution. **(C)** Normalized isoelectric point distribution. **(D)** Distribution of the lengths of identified peptides. **(E)** Distribution of protein sequence coverage distribution. **(F)** Peptide count distribution. **(G)** Protein ratio distribution between the 16HBE-GFP-*Plac9* and 16HBE-GFP lines.

Supplementary Figure 2 Volcano plot showing proteins differentially expressed between two cell lines (16HBE-GFP-*Plac9*/16HBE-GFP). Proteins with significantly different expression levels (≥1.2-fold, *p* < 0.05) between the cell lines were marked pink in the top right and left quadrants.

Supplementary Figure 3 STRING protein-protein interaction network of 587 differentially expressed proteins. A comprehensive description of protein-protein interactions in the PLAC9-overexpressed 16HBE cell line. The network nodes represent regulated proteins. Seven differently colored lines represent the seven types of evidence used in predicting the associations. The lines indicate the following: red, fusion evidence; green, neighborhood evidence; blue, co-occurrence evidence; purple, experimental evidence; yellow, text mining evidence; light blue, database evidence; black, co-expression evidence. The minimum required interaction score was the highest (0.900). The structure previews are displayed inside the network bubbles. All the disconnected nodes in the network were hidden. The details of this figure are presented in **Table S4**.

## Abbreviations

AEC, Airway epithelial cells; AGC, automatic gain control; BCA, bicinchoninic acid; BMAL1, brain and muscle aryl hydrocarbon receptor nuclear translocator-like 1; BRMS1, breast cancer metastasis suppressor 1; CDK, cyclin-dependent kinase; COPD, chronic obstructive pulmonary disease; DMEM, Dulbecco’s modified Eagle Medium; DMSO, dimethyl sulfoxide; EMT, epithelial-mesenchymal transition; FA, formic acid; FBS, fetal bovine serum; FITC, fluorescein isothiocyanate; GAPDH, Glyceraldehyde 3-phosphate dehydrogenase; GJD3, Gap Junction Protein Delta 3; GO, gene ontology; HCD, higher-energy collisional dissociation; IT, ion injection; iTRAQ, isobaric tag for relative and absolute quantification; KAAS, KEGG Automatic Annotation Server; KEGG, Kyoto Encyclopedia of Genes and Genome; LCs, lung cancers; LZTR1; Leucine Zipper Like Transcription Regulator 1; MS, mass spectrometry; MS2, Tandem mass spectrometry; MTT, 3-(4,5-Dimethylthiazol-2-yl)-2,5-diphenyltetrazolium bromide; NC, nitrocellulose; PAGE, polyacrylamide gel electrophoresis; PBS, phosphate buffered saline; PI, propidium iodide; PLAC9, Placenta-specific protein 9; PMSF, Phenylmethanesulfonyl fluoride; RT-qPCR, quantitative real-time polymerase chain reaction; SD, standard deviation; SDS, sodium dodecyl sulfate.
