# Supplementary figures and images for: Proteomic Analysis Reveals That Placenta-Specific Protein 9 Inhibits Proliferation and Stimulates Motility of Human Bronchial Epithelial Cells
Source: Front Oncol. 2021 May 28;11:628480. doi: 10.3389/fonc.2021.628480 (PMC8194706; doi:10.3389/fonc.2021.628480)

Figure S1

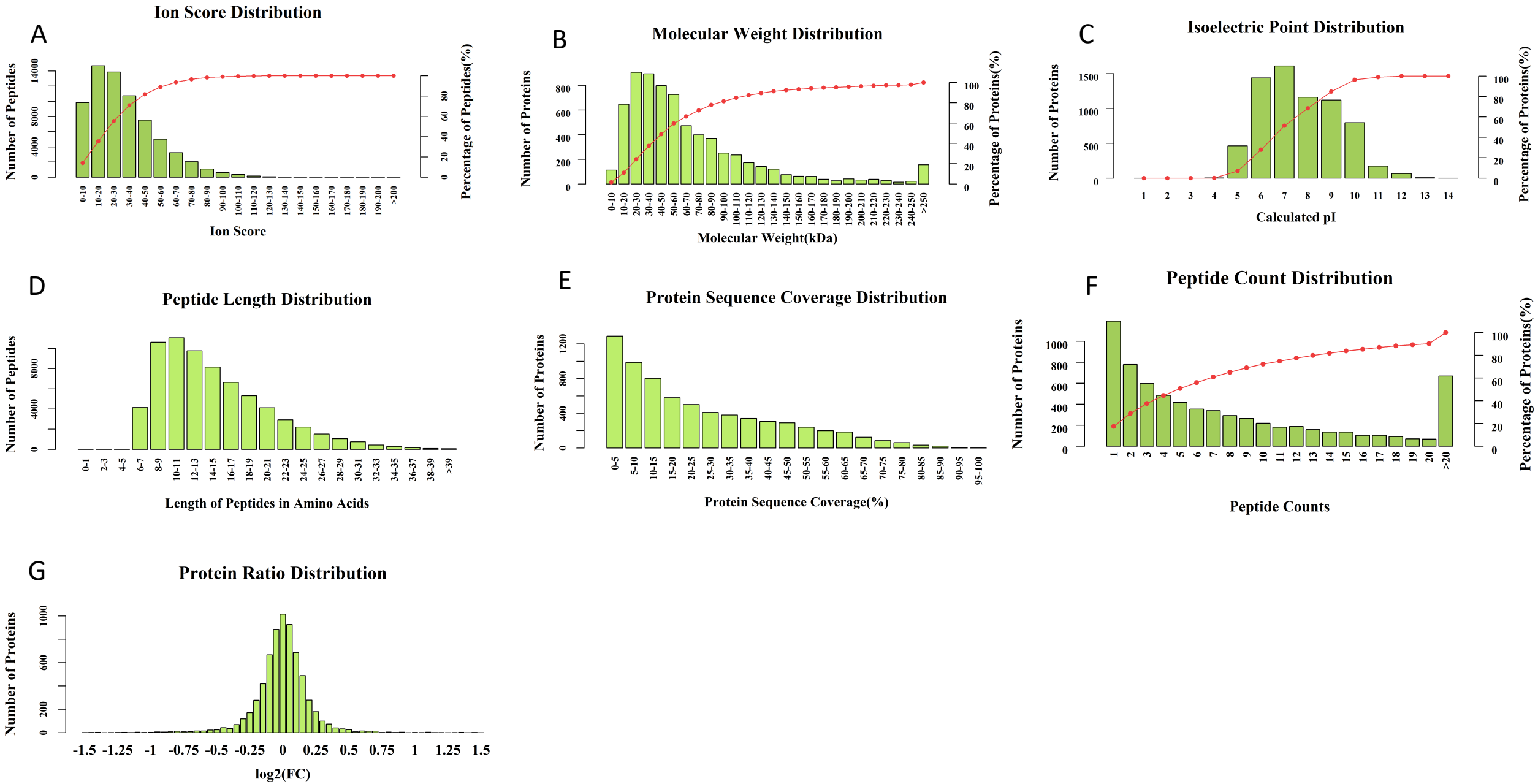

Supplement: Supplementary Figure 1 — Global characteristics of peptide properties identified in iTRAQ. (A) Normalized Mascot ion score distributions. (B) Normalized molecular weight distribution. (C) Normalized isoelectric point distribution. (D) Distribution of the lengths of identified peptides. (E) Distribution of protein sequence coverage distribution. (F) Peptide count distribution. (G) Protein ratio distribution between the 16HBE-GFP-Plac9 and 16HBE-GFP lines. [file DataSheet_1.zip › Supplementary Figure 1.PDF]

Figure S2

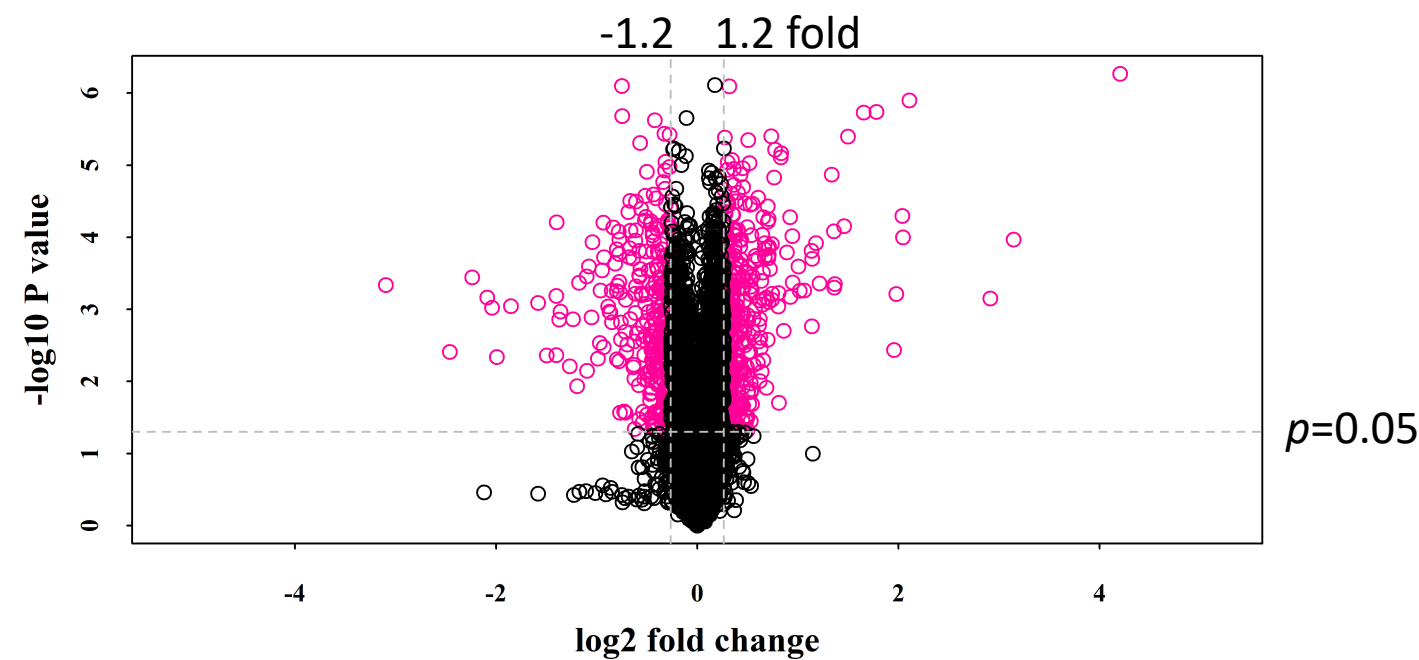

Supplement: Supplementary Figure 1 — Global characteristics of peptide properties identified in iTRAQ. (A) Normalized Mascot ion score distributions. (B) Normalized molecular weight distribution. (C) Normalized isoelectric point distribution. (D) Distribution of the lengths of identified peptides. (E) Distribution of protein sequence coverage distribution. (F) Peptide count distribution. (G) Protein ratio distribution between the 16HBE-GFP-Plac9 and 16HBE-GFP lines. [file DataSheet_1.zip › Supplementary Figure 2.PDF]
