# Supplementary material for: Proteomic Analysis Reveals That Placenta-Specific Protein 9 Inhibits Proliferation and Stimulates Motility of Human Bronchial Epithelial Cells
Source: Front Oncol. 2021 May 28;11:628480. doi: 10.3389/fonc.2021.628480 (PMC8194706; doi:10.3389/fonc.2021.628480)
Supplement: Supplementary Figure 1 — Global characteristics of peptide properties identified in iTRAQ. (A) Normalized Mascot ion score distributions. (B) Normalized molecular weight distribution. (C) Normalized isoelectric point distribution. (D) Distribution of the lengths of identified peptides. (E) Distribution of protein sequence coverage distribution. (F) Peptide count distribution. (G) Protein ratio distribution between the 16HBE-GFP-Plac9 and 16HBE-GFP lines. [file Supplementary Figure 3.PDF › Supplementary Figure 3.PDF]

Figure S3

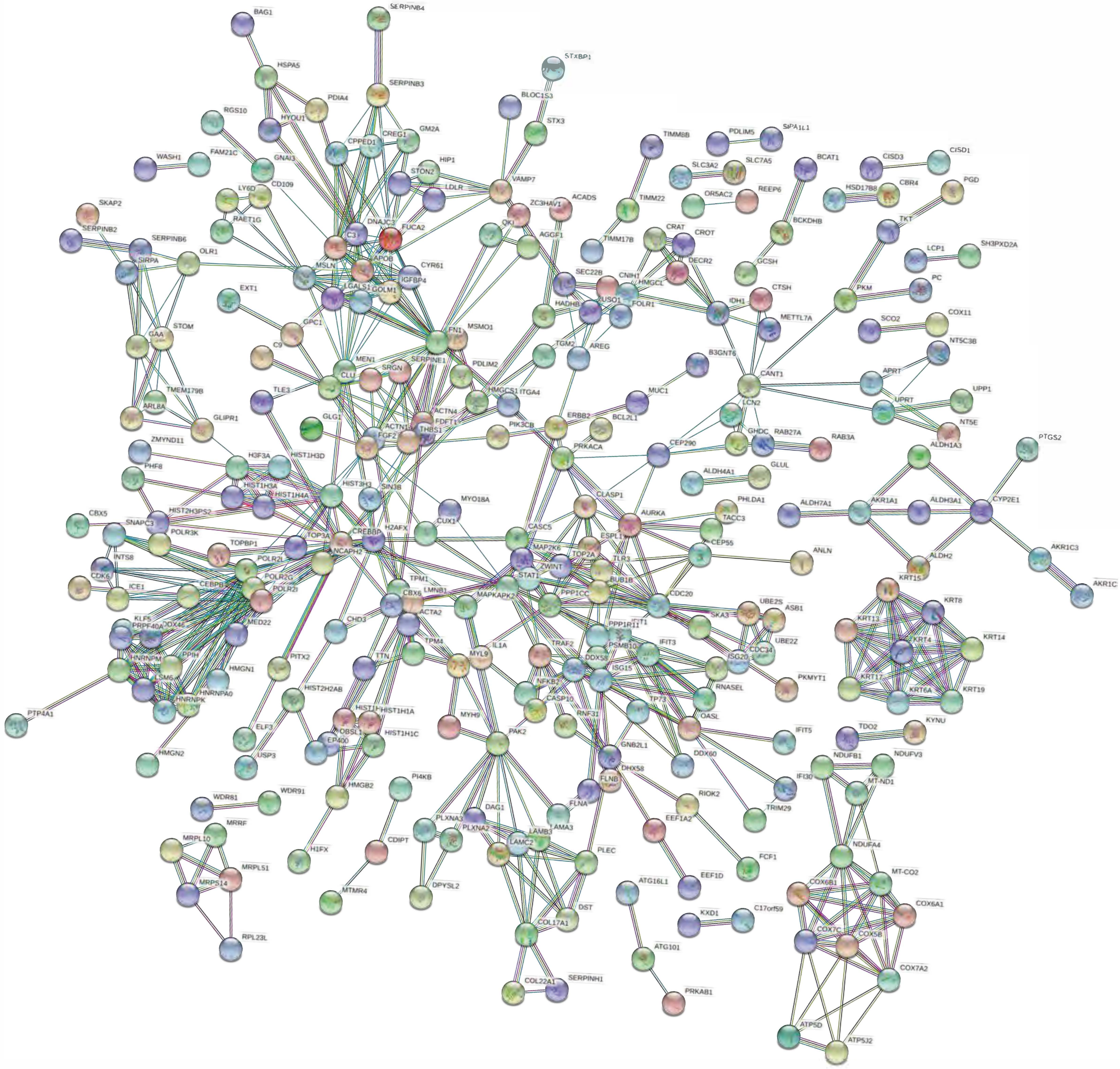

BAG1
SERPINB4
HSPA5
SERPINB3
STXBP1
BLOC1S3
STX3
PDIA4
HYOU1
RGS10
CPPED1
CREG1
GM2A
FAM21C
GNAI3
WASH1
HIP1
STON2
LDLR
VAMP7
LY6D
CD109
RAET1G
SKAP2
SERPINB2
SERPINB6
SIRPA
OLR1
C3
DNAJC3
FUCA2
ZC3HAV1
ACADS
QKI
AGGF1
APOB
MSLN
CYR61
LGALS1
IGFBP4
GOLM1
EXT1
GAA
STOM
GPC1
C9
FN1
MSMO1
MEN1
SRGN
SERPINE1
CLU
PDLIM2
TLE3
TMEM179B
ARL8A
GLIPR1
GLG1
ACTN4
FDFT1
ACTN1
THBS1
FGF2
HMGCS1
ITGA4
PIK3CB
ERBB2
BCL2L1
MUC1
ZMYND11
PHF8
H3F3A
HIST1H3D
HIST1H3A
HIST1H4A
HIST3H3
SIN3B
MYO18A
CBX5
HIST2H3PS2
SNAPC3
POLR3K
TOPBP1
TOP3A
CREBBP
H2AFX
CUX1
INTS8
NCAPH2
POLR2L
POLR2G
POLR2I
CDK6
ICE1
CEBPB
KLF5
PRPF40A
DDX46
MED22
HNRNPM
PPIH
HMGN1
HNRNPA0
HNRNPK
PTP4A1
HMGN2
TPM1
CBX6
LMNB1
ACTA2
CHD3
MAPKAPK2
TPM4
TTN
MYL9
IL1A
PITX2
HIST2H2AB
HIST1H1A
HIST1H1C
OBSL1
EP400
ELF3
USP3
MYH9
PAK2
HMGB2
PI4KB
H1FX
CDIPT
MTMR4
PLXNA3
PLXNA2
DPYSL2
DAG1
LAMB3
LAMC2
LAMA3
FLNA
PLEC
DST
COL17A1
COL22A1
SERPINH1
WDR81
WDR91
MRRF
MRPL10
MRPL51
MRPS14
RPL23L
TIMM8B
PDLIM5
SPATA1L1
SLC3A2
SLC7A5
BCAT1
CISD3
CISD1
HSD17B8
CBR4
PGD
OR5AC2
REEP6
TIMM22
TIMM17B
CRAT
CROT
BCKDHB
TKT
LCP1
SH3PXD2A
DECR2
SEC22B
CNIH1
HMGCL
HADHB
CUSO1
FOLR1
IDH1
CTSH
GCSH
PKM
PC
TGM2
AREG
METTL7A
SCO2
COX11
NT5C3B
B3GNT6
CANT1
APRT
UPRT
UPP1
LCN2
GHDC
RAB27A
RAB3A
NT5E
CEP290
PRKACA
ALDH4A1
GLUL
ALDH1A3
PTGS2
CLASP1
AURKA
PHLDA1
TACC3
ALDH7A1
AKR1A1
ALDH3A1
CYP2E1
CASC5
MAP2K6
ESPL1
TOP2A
TLR3
CEP55
ANLN
ZWINT
STAT1
BUB1B
PPP1CC
CDC20
AKR1C3
ALDH2
AKR1C
KRT15
KRT8
KRT13
KRT4
KRT14
KRT17
KRT6A
KRT19
SKA3
UBE2S
ASB1
UBE2Z
CDC34
ISG20
PPP1R11
IFIT1
PSMB10
IFIT3
TRAF2
DDX58
ISG15
NFKB2
CASP10
RNF31
TP73
RNASEL
OASL
PKMYT1
IFIT5
TDO2
KYNU
NDUFB1
NDUFV3
MT-ND1
GNB2L1
DHX58
FLNB
DDX60
IFI30
TRIM29
RIOK2
EEF1A2
EEF1D
FCF1
ATG16L1
ATG101
PRKAB1
KXD1
C17orf59
NDUFA4
MT-CO2
COX6B1
COX6A1
COX7C
COX5B
COX7A2
ATP5D
ATP5J2
